# Quality Control Methods for *Aedes albopictus* Sterile Male Transportation

**DOI:** 10.3390/insects13020179

**Published:** 2022-02-09

**Authors:** Georgios D. Mastronikolos, Apostolos Kapranas, George K. Balatsos, Charalampos Ioannou, Dimitrios P. Papachristos, Panagiotis G. Milonas, Arianna Puggioli, Igor Pajović, Dušan Petrić, Romeo Bellini, Antonios Michaelakis, Nikos T. Papadopoulos

**Affiliations:** 1 Laboratory of Entomology Department of Agriculture, Crop Production and Rural Environment, University of Thessaly, 38446 Volos, Greece; mastronikgeo@ymail.com (G.D.M.); ioannoubabis@yahoo.com (C.I.); 2 Scientific Directorate of Entomology and Agricultural Zoology, Benaki Phytopathological Institute, 14561 Kifissia, Greece; akapranas@agro.auth.gr (A.K.); g.balatsos@bpi.gr (G.K.B.); d.papachristos@bpi.gr (D.P.P.); p.milonas@bpi.gr (P.G.M.); a.michaelakis@bpi.gr (A.M.); 3 Laboratory of Applied Zoology and Parasitology, School of Agriculture, Aristotle University of Thessaloniki, 54124 Thessaloniki, Greece; 4 Centro Agricoltura Ambiente “G. Nicoli”, 40014 Crevalcore, Italy; apuggioli@caa.it (A.P.); rbellini@caa.it (R.B.); 5 Biotechnical Faculty, University of Montenegro, 81000 Podgorica, Montenegro; pajovicb.igor@gmail.com; 6 Faculty of Agriculture, University of Novi Sad, 21000 Novi Sad, Serbia; dusan.petric@polj.uns.ac.rs

**Keywords:** Sterile Insect Releases Technique, mosquito control, mass rearing, shipping, invasive mosquito species

## Abstract

**Simple Summary:**

The Sterile Insect Release Technique (SIT) is currently considered an environmentally friendly method to control populations of *Aedes albopictus*, an invasive mosquito species in Europe and elsewhere. Assessing the quality of mass reared, sterilized males that are transported to release sites is of the utmost importance for the success of the SIT programs. The current paper presents a series of quality control (QC) tests that have been conducted at the mass rearing facility in Italy and at delivery points in Greece and Montenegro to assess the impact of mass rearing, sterilization, and shipping on survival during transportation, longevity, flight ability and mating performance. Overall, our results reveal the detrimental effects of a long transportation period on the sterile male *Ae. albopictus* and provide a series of reliable QC tests to be considered in future SIT operations.

**Abstract:**

Genetic based mosquito control methods have been gaining ground in recent years for their potential to achieve effective suppression or replacement of vector populations without hampering environments or causing any public health risk. These methods require the mass rearing of the target species in large facilities sized to produce millions of sterile males, as already well established for a number of insects of agricultural importance. Assessing the performance of released males in Sterile Insect Technique (SIT) control programs is of the utmost importance for the success of the operation. Besides the negative effects of mass rearing and sterilization, the handling of sterilized insects and shipment to distant areas may also negatively impact the quality of sterilized males. The aim of the current study was to design and executive quality control (QC) tests for sterilized *Aedes albopictus* (Asian tiger mosquito) males delivered by air shipment from the mass production facility located in Italy to Greece and Montenegro field release sites. Mass reared mosquito strains were based on biological materials received from Italy, Greece and Montenegro. Tests conducted at the mass rearing facility before transportation revealed a rather high residual female contamination following mechanical sex separation (approximately 1.5% females, regardless of the mosquito strain). Irradiated males of all three mosquito strains induced high levels of sterility to females. Shipment lasting approximately 24 h resulted in approximately 15% mortality, while when shipment lasted nearly two days this increased to almost 40%. The flight ability of sterilized males following one day transportation time was satisfactory (over 60%). The response of sterile males to food and water starvation was comparable and slightly lower than that of wild non-transported males. Longevity of sterile males was shorter than that of wild counterparts and it seems it was not affected by mating to wild females. Both mating propensity and mating competitiveness for wild virgin females was higher for the wild, control males compared to the sterile, transported ones. Overall, the performance of sterile male *Ae. albopictus* delivered from the mass rearing facility of Italy to Greece in approximately 24 h was satisfactory. Transportation lasting two days or longer incurred detrimental effects on males, which called into question the outcome of the SIT release programs. In conclusion, our results demonstrate the need of quality control procedures, especially when sterile male production facilities are not near to the releasing point. Transportation could be a serious drawback for the implementation of Sterile Insect Releases and, consequently, it is important to establish an efficient and fast transportation of sterilized males in advance.

## 1. Introduction

The Sterile Insect Technique (SIT) is an environmentally friendly method for insect population management (suppression, eradication, and prevention of establishment) that involves the mass rearing and release of large numbers of sterilized males in the wild [1,2,3,4,5]. Sterile males’ mate with wild feral females that subsequently lay infertile eggs, gradually decreasing the reproduction rates of wild populations. Hence, under the assumption that enough competitive sterile males are released for several weeks, the local wild population can be substantially suppressed and even eradicated [6,7,8].

Apparently, the success of an SIT project depends on the ratio of released sterile males to wild males and, if the quality of the sterile males is low, then the efficacy of SIT is reduced [9,10]. Most SIT programs employ releases of large quantities of sterilized males that outnumber the wild males (“overflooding ratios”). The costs and labor associated with insect production would be prohibitive for many operations and a balance must be reached which ensures that enough sterile males are produced and released at the lowest cost possible [8,11]. Additionally, the negative effects of radiosterilization on mating capacity and on longevity can be minimized by adjusting the timing of irradiation and the dose [9,12,13]. Earlier studies in fruit flies (Diptera: Tephritidae) have shown that both irradiation at high doses to achieve high rates of sterility [14,15,16] and mass rearing reduce the sexual competitiveness of sterile males in contrast to wild ones [15,17]. Besides the negative effects of mass production and sterilization on the quality of sterile males, transportation to release sites may induce an additional stress and, hence, deterioration of the quality. Several aspects of transportation, such as the means of transportation, duration and ambient condition need to be considered, in order to disentangle the possible negative effects on the performance of delivered males.

The quality attributes of the released sterile males include the ability to survive and disperse in the wild, locate and find a mate, perform sexual courting, mate and transfer sperm and ultimately induce sterility and refractoriness to mated/re-mated females [18]. These quality attributes can be assessed during mass rearing and before their release, and indeed there are several developed protocols for different target species available [11,19,20,21]. Most of the established protocols concern species with a long history in SIT, such as fruit flies, the tsetse fly (*Glossina austeni* Newst.), and the screw worm (*Cochliomyia hominivorax* Coquerel) [1,2,3,4,5].

Since its successful use for many agricultural pests, SIT is now proposed and evaluated to address pests of importance in public health, such as mosquitoes [22,23]. Past efforts for the control of mosquitoes using the SIT partially failed because of the low competitiveness and general performance of the sterile males compared to the wild ones [24,25,26]. Recent SIT efforts target invasive mosquito species, such as *Aedes* (Stegomyia) *albopictus* (Skuse) and *Aedes* (Stegomyia) *aegypti* (Linnaeus), which are major vectors of diseases, such as the Zika and Dengue virus [27,28]. New techniques in mass rearing and sterilization have been developed that substantially improve the quality of sterile male mosquitoes.

Standard quality control tests (QC) have been developed for numerous insect species where SIT has been applied [29]. Quality control tests may include traits to assess proportion of emergence, flight ability, stress resistance, longevity and mating competitiveness with a goal to predict the performance of the sterile insects in the wild. The quality of the sterile mosquitoes can be assessed in laboratory, semi-field and field conditions [30,31]. Standard QC procedures in most advanced SIT programs include the insect’s ability to fly out of simple tube devices [32,33,34,35]. Balestrino et al. [29] developed and validated new tools designed to infer the quality of *Ae. albopictus* sterile males through the observation of their flight ability after stress treatments (different aspiration powers and times) in a ‘flight organ device’. Results have shown a correlation between flight ability, survival and mating capacity. Culbert et al. [36] improved the device, which was tested on *Ae. aegypti* and *Ae. albopictus*, showing enough sensitivity to detect the effects of irradiation, chilling, and compaction.

Since there is lack of information on the quality of males transported from the rearing facilities by air cargo shipment to distant areas and often countries where they are released [37], the current paper presents a list of tests to assess the quality of transported sterilized males. More specifically, we describe the experimental procedures that test whether transportation under realistic SIT operation programs affect the quality of the released males.

## 2. Materials and Methods

**Figure 1:**
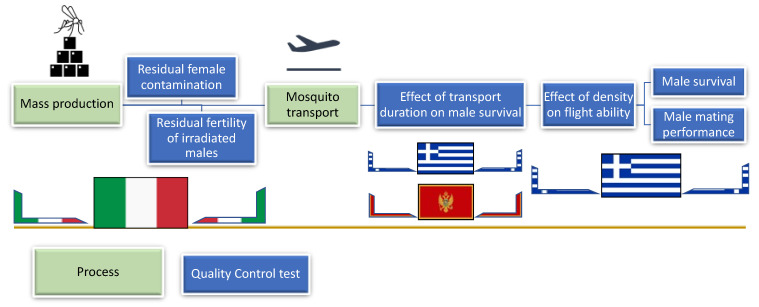
Flow diagram outlining of the processes from mass production to transport and the respective Quality Control (QC) tests performed. The flags of Italy, Greece and Montenegro indicate the activities conducted in respective states.

Several QC tests were organized and performed in Italy (mass production facility, Crevalcore, Bologna), Greece and Montenegro (Figure 1). Initially, we assessed the residual fertility of produced sterilized males (Italy) and then we evaluated the effect of transportation on male survival and flight ability (Greece, Montenegro). After finding the optimum transportation density we further evaluated (Greece): (a) the effect of mating, food and water stress on male survival; and (b) the male mating performance. Due to different custom procedures between European Union (EU) and non-EU countries, the duration of transportation and its effect on mortality rate was also recorded for transportations from Italy to Greece, (EU country) and to Montenegro (non-EU country).

### 2.1. Mosquito Strains

#### 2.1.1. Mass Rearing of *Aedes albopictus*

All males used in these trials originated from *Ae. albopictus* field collected eggs from Greece (Vravrona), Montenegro (Radovići) and Italy (Rimini) (GR, MNE and RER strain, accordingly). Colonies were established in the BSL3 laboratory of the Medical and Veterinary Department of the Environmental and Agriculture Centre “G. Nicoli” in Crevalcore (CAA) (Bologna, Italy). Adult mosquitoes were reared at standard conditions (28 ± 1 °C, 85 ± 5 % RH, 14:10 h L:D photoperiod) in plexiglas cages (40 cm× 40 cm× 40 cm) and were fed constantly on a 10% sucrose solution [38]. Females were offered fresh swine blood every week, using a Hemotek^®^ membrane feeding system (thermostat-controlled device by Hemotek Ltd., Great Harwood Business Zone, Blackburn) [38]. Eggs laid on wet filter paper were removed from the adult plexiglass cages and placed in sealed plastic boxes to permit embryonation. When needed, larvae were obtained using standardized hatching procedures and reared at standard conditions inside white plastic trays (41 cm× 31 cm× 11 cm) containing 2 L of deionized water, 4000 larvae fed with 2.0 mg/larva of the International Atomic Energy Agency (IAEA, Vienna, Austria) standard diet integrated with brewer yeast (IAEA-BY) administered daily for four days after hatching [29].

#### 2.1.2. *Aedes albopictus* Greek Population (Control)

In addition to the males which were transported and used in a series of experiments, we also used a wild Greek population which originated from eggs collected in Athens using ovitraps (Vravrona strain, the same used for mass production in Italy; Bellini et al. [39] as a non-treated control (neither irradiated, nor transported). Egg batches were collected daily and kept sealed in the laboratory of the Benaki Phytopathological Institute (BPI) (Kifissia, Greece) or transported to the laboratory of Agricultural Zoology at the University of Thessaly (UTH) (Volos, Greece, driving distance 3 h from BPI). Before trials, eggs were submerged in stale tap mineral water for hatching and rearing [37,40,41,42]. The flight ability tests were conducted at BPI, whilst for the survival and mating performance tests (effect of mating, food and water stress on male survival and male mating performance) eggs (in oviposition substrates) were transported to UTH and reared there to serve as the respective controls in various tests.

### 2.2. Sexing and Irradiation

Sex separation (male pupae) from the three mass reared populations (GR, MNE, RER), was based on pupae size and was conducted using metal sieves with square meshes of 1400 µm [38,43], 24–30 h from the beginning of pupation. The collected pupae were aged for an additional 24 h and then divided into batches of 5000 pupae and transferred into plastic containers of 1000 mL capacity, filled with 500 mL of tap water. These containers were used to transport the pupae to the Medical Physics Department of the St. Anna Hospital (Cona, Ferrara, Italy) for irradiation and, afterwards, back to the laboratory for adult emergence and packaging [44]. During the night before irradiation, as well as during transportation to irradiation, the pupae were maintained in a thermal insulated plastic container to maintain a temperature of about 20–22 °C. Few adult males may emerge during transportation of the pupae to/from the irradiation.

Irradiation of pupae were performed by using an IBL 437 irradiator (CIS Bio International, Bagnols-sur-Ceze, France) equipped with a 50.9 TBq Cs-137 linear source with a central dose rate of 1.8 Gy/min [44]. Pupal batches were transferred with water into petri dishes (12 cm diameter) and were piled inside a dedicated canister for irradiation. The gamma ray dose of 35 Gy was administered to the pupae, at the time aged about 24–48 h. The dose distribution inside the basket has been recently checked using GAFCHROMIC EBT3 self-developing dosimetry films (International Specialty Products, Wayne, NJ, USA).

For the estimation of the sex ratio of the selected pupae, a sample of 300 pupae were sexed individually under a stereomicroscope using morphological characters [45].

### 2.3. Residual Fertility of Irradiated Males

The residual fertility of all strains was checked under laboratory conditions at CAA. Irradiated male pupae were placed in cages with virgin, non-irradiated conspecific female pupae of the same strain. As a control, non-irradiated male pupae from each strain were also caged with conspecific virgin females. The male and female pupae used for these experiments were checked and sexed individually under a stereomicroscope [46,47].

Different replicates were performed per strain and gonotrophic cycle using cages with 100 males and 100 females with constant access to a 10% sucrose solution. Blood meals were offered to the females on day 7 and day 14 from adult emergence (two gonotrophic cycles). Eggs laid on filter papers were collected five days after blood feeding, left to dry slowly and stored at high humidity for at least one week before hatching. The eggs collected were counted under a stereomicroscope and hatched overnight in bacterial broth solution [44]. After submersion, the egg papers were checked under the stereomicroscope to count the hatched and unhatched eggs.

### 2.4. Direct Effects of Transportation on Sterile Males

#### 2.4.1. Direct Effect of the Duration of Transportation on Survival

Transport from the CAA mass production facility (Crevalcore, Italy) to Athens (Greece) and Podgorica (Montenegro) of sterilized males of the respective strains was conducted by air shipment. Adults were sent in chilled foam boxes (around 1500 sterilized males/box: temperature 8–14 °C) covered with net and a cotton fiber wet with a 10% sugar solution and ice packs [37]. To evaluate the direct effect of transportation duration, upon arrival we counted the number of dead males per shipment in both countries. The transportation period for Montenegro was May–June 2018 and for Greece August–September 2018.

#### 2.4.2. Effect of Density during Transportation on Flight Ability

Sterilized males (GR strain) were transported from Italy to Greece in three different densities: 1200, 1500 and 1900 males/chilled foam box. As the control, non-irradiated and non-transported males of the same age (3–4 days) originating from the Greek control colony maintained at the BPI facility were used. Upon arrival, sterilized males were transferred to constant laboratory conditions (25 ± 2 °C, 80% RH) in the BPI laboratory and fed ad libitum with 10% sugar solution for five hours. The control males were kept in the same conditions.

Flight ability tests were conducted using the aspirator device and the methodology described by Balestrino et al. [29]. Twenty males from each of the four density regimes and the laboratory control males were transferred to each suction tube. The aspiration time was set at 30 min, while 10 and 15 replicates were performed for sterilized and control males, respectively. This stress test can measure possible biological stress conditions and it was selected as an easy and rapid method compared to other methods [36].

### 2.5. Post Transportation Survival and Mating Performance

Experiments were conducted at constant conditions (25 °C, 65% RH, 14:10 L:D) in the laboratory of Entomology and Agricultural Zoology at UTH. The photophase was set from 07:00 to 21:00 with 45 min increasing/decreasing light intensity at dawn and dusk respectively.

#### 2.5.1. Effect of Mating, Food and Water Stress on Male Survival

Effect of Mating on Male Longevity

To determine the effect of mating on male longevity, both virgin wild and sterilized males were allowed to mate with wild virgin females. To do so, 40 either sterilized or wild males were placed in a BugDorm^®^ cage together with 40 virgin females. Three such cages (replicates) were set up for each male type. Adults of both sexes were confined together for six days to allow mating. On the seventh day, 50 randomly chosen males of each category (from all three replicates) were transferred with an aspirator into individual cages, having access to a 5% sugar solution. Fifty males of both types (wild and sterilized) having no access to females and kept in similar crowding density conditions (80 males per cage) for six days were included in our trials as the respective unmated controls. Individual adult cages were constructed of a transparent, 400 mL capacity plastic cup that was placed upside down and fixed on the lid of a 9 cm diameter petri dish. A side window (3 cm × 5 cm) served for ventilation, while a small hole on the top of the cage provided the entrance to the cage. Adult food (5% sugar solution) was provided by a cotton wick that went through the base of the cage to the underneath base of the petri dish. Male mortality was recorded daily until the death of the last individual.

#### 2.5.2. Effect of Food and Water Starvation on Adult Survival Rates

The following three treatments were tested: (a) food starvation (males kept with only water), (b) food and water starvation (neither food nor water offered to males), and (c) control (both food and water offered ad libitum to males). Sterilized males were placed in individual cages (see above) upon arrival in the laboratory. Wild males of the same age (four days old) were placed in the same cages as well. Sterilized and wild males were randomly assigned to one of the three treatments (50 individuals per treatment). Mortality was recorded twice a day until the death of the last individual.

### 2.6. Male Mating Performance

#### 2.6.1. Mating Propensity and Daily Patterns of Mating Frequency of Wild and Sterilized Males

To choose the appropriate time interval of the day to perform both mating propensity and mating competitiveness tests we recorded the daily patterns of mating in a “no choice” experimental set up. Before being tested, batches of virgin sterilized males, virgin wild males and virgin wild females were kept separately in standard BugDorm-4F2222 cages at approximately 80 adults per cage, and provided with a 5% sugar solution. All adult mosquitoes were reproductively mature (6–12 days old). Mating arenas consisted of a transparent Plexiglass^®^ cage (15 cm × 15 cm × 15 cm, custom made) with amble adult food (sucrose solution 5%). In each mating arena, one female was placed in the plexiglass arena the evening before the test for acclimation to the environment and one male was introduced just before the start of the observation. The total number of copulations, as well as the duration and the latency of the first copulation (time until the first copulation) and of every next one (from the end time of the previous copulation until the start time of the new one), were observed for three time periods set during the photophase (08:00–12:00, 12:01–16:00, 16:01–20:00). For each time period we conducted 30 replicates. To compare the mating propensity between the wild and sterile males we combined the data of the three time periods for both mating success and mating characteristics (i.e., latency and mating duration time).

#### 2.6.2. Male Mating Competitiveness

The mating competitiveness of the sterile vs wild males were conducted using five to ten days old virgin males. Each test involved virgin sterile males, virgin wild males and virgin wild females at a ratio of 1:1:1. Tests were conducted in the Plexiglass mating arenas (see above) from 18:00 to 20:00 based on earlier observation regarding daily patterns of the mating activity. Males were marked with a florescent powder before mating. We ran 98 replicates and mattings were visually recorded by experienced personnel. In half of the replicates the sterile male was colored with powder non-toxic dye and in the other half of them the wild male was colored. Hence, we conducted (a) 49 replicates with one female/one non-colored sterile male/one colored wild male and (b) 49 replicates with one female/one colored sterile male/one non-colored wild male. The wild female was set in the testing arena one day before the test day for acclimation and the males (sterile and wild) were introduced together at the start of the experiment. We recorded the duration and the latency to first mating (time between the introduction of the males in the arena until the first mating) and for every next copulation formed (end time of the previous copulation until the start time of a new copulation with the same individual).

### 2.7. Statistical Analysis

General linear model (GLM) followed by the Tukey’s HSD test for post-hoc comparisons of means was used to test differences among strains (GR, MNE, RER) in residual female contamination following the mechanical sex separation of the pupae. Likewise, the effect of the mosquito strain, the level of irradiation and the gonotrophic cycle on egg hatch rate was investigated with GLM. Residual fertility rates were corrected with Abbott’s correction formula [48]. One-way ANOVA was used to test the effect of mosquito density during transportation on flight ability. Survival analysis was considered to test whether the response (time to death) of wild and sterilized males to food and water starvation was differed. The log-rank test (Mantel–Cox) was used to compare the two groups of males. The effects of male type (wild, sterilized) and mating (mated, non-mated) on male longevity was assessed using the Cox proportional hazards model. Pairwise comparisons were conducted using the log rank (Mantel–Cox) test. Chi-square tests was used to separate rates of matings between wild and sterilized males in no choice mating tests and in choice mating tests. For all analyses the significance level was set at α = 0.05. Data analysis was performed using IBM SPSS 25 (IBM Corp., Armonk, NY). The Ms Excel and ggplot in RStudio v1.1.453 (RStudio 2012, R Foundation of Statistical Computing, Vienna, Austria) were used to plot graphs.

## 3. Results

### 3.1. Residual Female Contamination

The female contamination observed in the male pupae, following mechanical sex separation, did not differ as a function of the different strain used (F_2, 14_ = 0.425, *p* = 0.662). The mean percentage (±SE) of the residual presence of females obtained with the RER, GR and MNE strains was equal to 1.62% (±0.32), 1.25% (±0.26) and 1.18% (±0.39) of the total number of pupae selected, respectively.

### 3.2. Male Residual Fertility

No difference in the fertility of *Ae. albopictus* females as a function of the strain (*F*_2, 81_ = 1.5; *p* = 0.23) and gonotrophic cycle considered (F_1, 81_= 0.03; *p* = 0.86) were detected. However, irradiation of males had a significant effect on female fertility (*F*_1, 81_ = 3508; *p* < 0.001). The overall mean (± SD) residual fertility measured by egg hatch was significantly lower (0.008 ± 0.007) than the recorded fertility in the untreated control group (0.831 ± 0.089) (Table 1).

### 3.3. Direct Effect of the Duration of Transportation on Sterilized Male Survival

The time of transportation required to deliver males to Montenegro was approximately two days, while the delivery to Greece was approximately one day, and this led to a significant higher mortality rate (*t*-test, *p* < 0.05) in the male mosquitoes transported to Montenegro (Table 2).

### 3.4. Effect of Density during Transportation on Flight Ability of Sterilized Males

**Figure 2:**
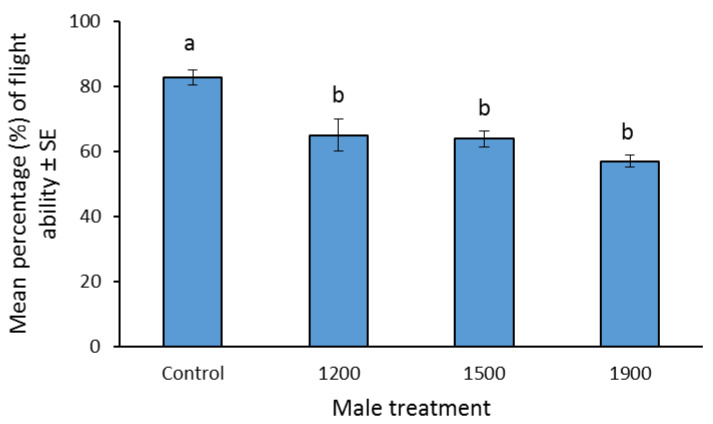
Flight ability of sterilized *Aedes albopictus* males shipped at different densities and the wild non-shipped, fertile males of the control population. Different letters indicate significant differences (Tukey’s HSD test, *p* < 0.005).

The proportion of males initiating flight was higher in control males compared to sterilized ones, regardless of the density of mosquitos during transportation (*F*_3, 41_ = 16.2, *p* < 0.001), while no differences were found among the three densities of transportation (Figure 2, Tukeys’ HSD tests, *p* > 0.05).

### 3.5. Effect of Mating, Food and Water Stress on Male Survival Post Transportation

Both wild and sterilized males had similar life spans under the conditions of food starvation (log rank test, *p* > 0.05) (Table 3). However, wild males live longer than sterilized ones under both food and water starvation (log-rank test, *p* < 0.05) (Table 4).

**Figure 3:**
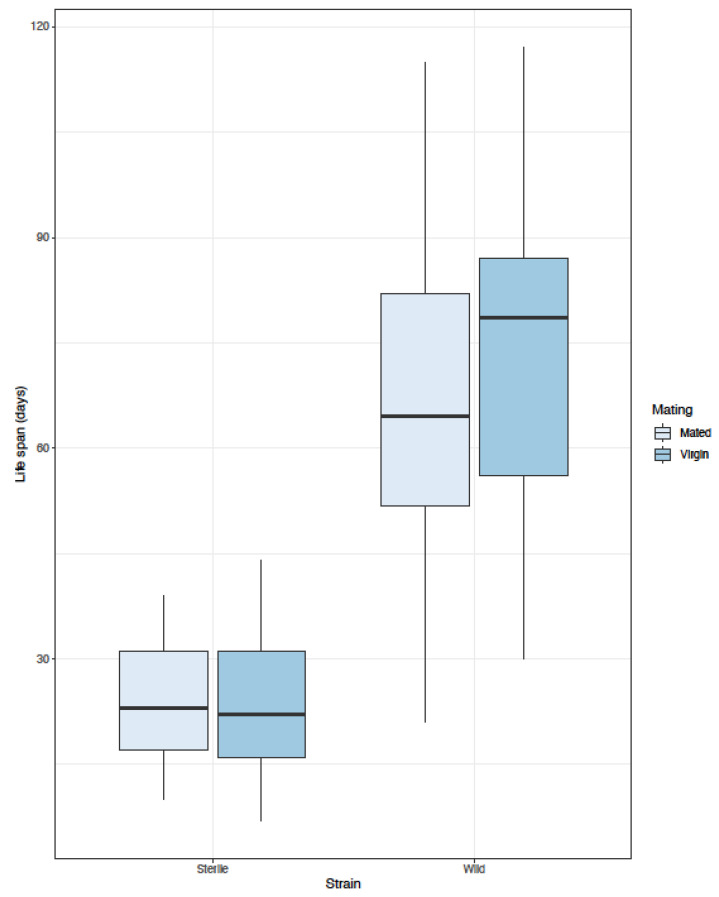
Box plots depicting the effect of male type (wild or sterilized) and mating status (mated or virgin) on longevity of males.

Regardless of mating status, the lifespan of wild males was no longer than that of the sterilized ones (Table 5 and Figure 3). On the other hand, mating did not have an apparent effect on males’ longevity. Likewise, the interaction between male type and mating was not significant, indicating that the effects of mating were similar on the two male types.

### 3.6. Male Mating Performance Post Transportation

#### 3.6.1. Daily Patterns of Mating Frequency of Wild and Sterilized Males

**Figure 4:**
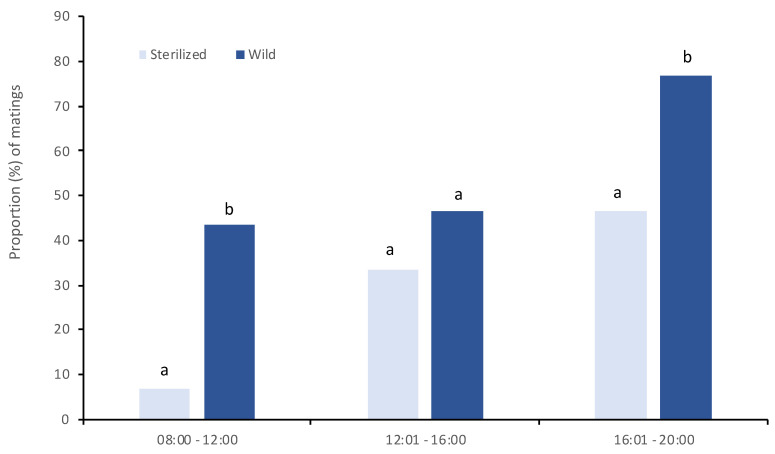
Daily patterns of mating for wild and sterilized males when wild virgin females were offered in a no choice experimental set up. Different letters indicate significant differences within each time interval (*p* < 0.05).

Both wild and sterilized males exhibited higher rates of mating in late afternoon and evening hours (Figure 4). Wild males mated at higher rates early in the morning and late in the afternoon compared to the sterilized ones (x^2^ =10.75 and 5.71 respectively, df = 1, *p* < 0.05). Mating proportions were similar between wild and sterilized males at noon (x^2^ = 1.11, df = 1, *p* > 0.05). Overall, wild males mated at higher rates than sterilized ones, independently of the time period.

**Figure 5:**
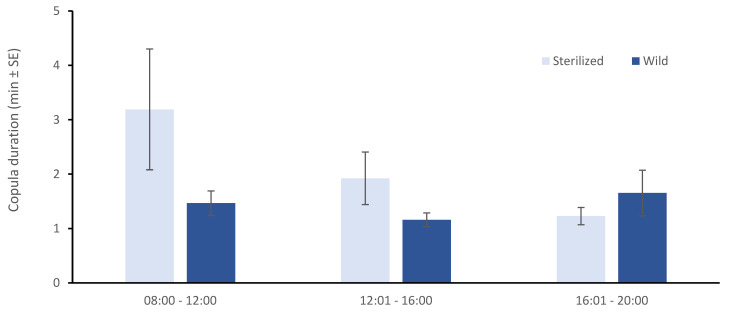
Effect of time of the day on copulation duration for wild and sterilized males when wild virgin females were offered in a no choice experimental set up.

The duration of mating for both wild and sterilized males and for all three time periods is given in Figure 5. Despite a trend for longer mating duration during morning hours for the sterilized males, no differences were reported when all three time intervals were included in the analysis (*t*-test = 0.049, df = 178, *p* < 0.05). 

#### 3.6.2. Mating Propensity

Combining data of the three time periods above, it was found that wild males mated at a higher (almost two-fold) proportion compared to sterilized ones under the no choice experimental set up. In total 90 replicates were considered for each male group. Fifty and 26 females out of 90 were mated with the wild and the sterile males, respectively (55% and 28% mating for each male group, respectively, Table 6). The latency to mate was substantially but not significantly longer for the sterilized males compared to wild ones. Likewise, the mating duration was similar in the two types of males.

#### 3.6.3. Mating Competitiveness

Mating with either a wild or a sterilized male was recorded in 71.4% of the test cages (98 in total). Virgin females mated at higher rates with wild non sterilized males compared to sterilized ones (Table 7). The latency to mate was shorter for the sterilized males compared to wild ones, while mating duration was similar for the two types of males.

## 4. Discussion

The quality of sterile males is an important parameter for the success of an SIT program. In this study we assessed some parameters contributing to the quality of sterile *Ae. albopictus* males. Currently there is much interest for managing urban populations of *Ae. albopictus* with SIT and our study is aligned with initiatives of the International Atomic Energy Agency (IAEA), the INFRAVEC2 project and AIM-COST Action CA17108 aiming to enhance the capacity to integrate SIT in the effective management of invasive *Aedes* mosquitoes. In this study different mosquito populations were sampled as eggs from the targeted areas, sent for mass rearing and sterilization in a specialized facility in Italy and tested in the trial areas in Greece and Montenegro. Hence, the effect of mass rearing sterilization and transportation have been considered in our trials. One important aspect for the SIT program is the sexing (separation of males from females) of insects. In our study, the mechanical sex separation method applied allowed a residual presence of females of around 1.35% (±0.21), which is still slightly higher than in the case of large SIT programs based on WHO [49]. Moreover, the impact of these released sterile females has been studied recently in Dumont et al. [50] when viruses are not circulating, releasing sterile females is not really an issue, while it is an issue when viruses (e.g., Dengue, or Chikungunya virus) are circulating.

The performance of the sterile males is influenced by a combination of factors: rearing conditions, sexing procedure, radiation dose and method, and packaging condition during transportation. The mass rearing of *Aedes* mosquitoes may induce developmental changes that affect body size, response to environmental stress and reduced male sexual performance and insemination ability [51,52,53,54]. *Aedes albopictus* colonies can maintain integrity and quality if there is sufficient genetic variation in mass rearing conditions resulting from optimized rearing conditions [55]. The optimal irradiation dose is determined experimentally by dose-response curves [44]. Our results confirm the high sterilization level obtained on adult mosquito males irradiated with a dose of 35 Gy at the pupal age of 24–40 h. The different European populations of *Ae. albopictus* tested have similar sensitivity to the gamma radiation dose used, without variation as a function of the different gonotrophic cycle of females considered. Hence, it seems that irradiation induces universal effects on different *Ae. albopictus* strains.

Shipping can also have a negative influence on the sterile insect quality [56]. Shipping requires the chilling and packaging of sterile males at high densities, which can have a marked influence on their quality [26,56]. Our findings indicate that transportation lasting two days or longer, such as the one from Italy to Montenegro, has a marked direct negative effect on the survival or sterile male mosquitoes, when compared to shorter transportation, such as the one from Italy to Greece (20–24 h). This result highlights the need for a harmonized process regarding the international framework for the trade of sterile male mosquitoes involving agreement among the involved authorities [57,58].

Moreover, transported sterile males results in a reduced flight ability compared to the wild ones, as assessed by flight apparatus used in similar trials [29]. On the other hand, density in the range of 1200–1900 males per container did not influence the survival rate and flight ability of shipped mosquitoes, which is positive for the logistics of SIT programs. Similarly, compaction did not significantly affect the survival of sterile *Anopheles arabiensis* males [59].

The sterile males shipped to Greece lived for a significantly shorter time than the wild ones at conditions of total starvation. This test serves as an index of nutritional reserves present in the males at after the time of release (FAO/IAEA/USDA 2019). On the other hand, provision of water, which is a more realistic scenario in natural conditions, led to equal survival rates of sterile and wild mosquitoes, indicating that, despite the stress caused by transportation, sterile males can still live long enough.

The interpretation of tests assessing the mating propensity (ability) of sterile male mosquitoes should consider their mating system (behavior) as well as the conditions of mass rearing. *Ae. albopictus* mating behavior is characterized by several rather small male aerial swarms where male competition take place and allows for female choice [60,61]. In our tests it was determined that wild males were more eager to mate than sterile males; however, this finding might not be representative of male performance when they encounter wild females in the field, because, in our experiments, females were reared in the laboratory and tests were conducted in confinement [62].

Nevertheless, when sterile mosquitoes directly competed with wild ones for mating to females, the latter were two times more successful. This can be in part attributed to sterilization, in part to mass rearing and in part to handling/shipping. The latency to mate was similar for non-sterile males when compared to sterile ones. The non-significant difference is attributed to high variability (range from 1 to 231 minutes). The selection for rapid mattings of male mosquitoes in high density mass-rearing conditions has been demonstrated earlier [62]. Under the artificial rearing conditions, the “domestication” effect could lead to major changes of behavioral and physiological traits. This phenomenon is well known when insects are mass produced under laboratory conditions which are different (high crowding) from those to which the species is adapted in the field [62] and references therein).

Previous studies on establishing quality control methods for sterile *Ae. albopictus* included flight tests with an aspirator device to assess tolerance to mechanical and desiccation stresses, as well as male mating capacity, however they did not directly compare the mating competitiveness of sterile vs wild males [27]. Our trials indicate that sterile male mosquitoes have a reduced performance when compared with non-sterilized males, but they should be interpreted with caution, especially in assessing factors, such as mate propensity/competitiveness. Competitiveness tests that are carried out in large field enclosures may be more representative of real field condition [63]. For *Ae. Albopictus*, a previous study [60] determined that the mean CIS index (capacity to induce sterility index or competitiveness index) in the field strongly varied in space and time, ranging from 0.02 to 0.37, which indicates that the sterile males were 3 to 50 times less competitive than the wild males. Madakacherry et al. [11] found that the sterile males were equally competitive with their unirradiated counterparts, and a 5:1 ratio was sufficient to reduce, but not eliminate, the fertility of the female populations, irrespective of cage size.

Previous studies and the results presented herein indicate that irradiated male *Ae. albopictus* can effectively compete against fertile males, indicating that both the dose rate and the life stage at which the males are irradiated are near optimal [21,44,64]. However, given the increasing interest in adopting the SIT technology against the tiger mosquito globally, and the importance of standardized quality control, there are still more studies needed on the performance of sterilized males on the quality traits that were outlined earlier. In this study, all trials were conducted in 2018, before the beginning of the weekly releases in Greece aiming to check the quality of irradiated sterile male mosquitoes [37]. Thus, it is important to stress that our quality control procedures took place not near the mass production facility but in the target area of an SIT pilot study. This might be more relevant to quality control studies conducted at the mass rearing facility, since they include additional stresses related to shipping and handling.

## 5. Conclusions

Results of the current study indicate clearly that it is challenging to manage massive releases when sterile male production facilities are too far from the releasing point, especially when transportation of sterile males lasts longer than 24 h by air travel. The COVID-19 pandemic and its effects on transportation (flights delays or cancelling) further highlights the importance of establishing an efficient and fast transportation and timely delivery of the sterilized males. Quality Control (QC) processes are needed to ensure the feasibility of SIT control when shipments of sterile males are required. Quality Control trials should be conducted before shipping (at the production facilities) and after transportation (at the release area) to precisely partition the effects of irradiation/rearing/handling and that of transportation on male performance. To accommodate sterile male releases in distant areas, the development of a single *Ae. albopictus* mass rearing strain should be considered. To establish such a generic strain, propagules (genetic material) from different countries should be included. Apparently, the compatibility of the new established strain against local SIT target populations should be evaluated with extensive quality control tests. In conclusion, in the current study laboratory quality control protocols for sterilized mosquito males were applied to address, for the first time, the effects of shipment in the target area on the performance of sterilized males. The results provide useful baseline information for the implementation of SIT when sterile male production facilities are not near to the releasing point and shipment is required.

## Figures and Tables

**Table 1:** Residual fertility (Abbott’s corrected) of eggs laid by *Aedes albopictus* females mated with conspecific males irradiated as pupae at 35 Gy. Strains: Greece (GR), Montenegro (MNE) and Italy (RER).

Strain	Trials	N	Total No Eggs Checked	Average Residual Fertility (Eggs Hatched/Eggs Oviposited + SD)	
				Control	Irradiated
RER	1	10	30,927	0.834 ± 0.059	0.007 ± 0.008
	2	10	28,978	0.842 ± 0.096	0.006 ± 0.003
GR	1	5	6378	0.820 ± 0.187	0.013 ± 0.012
	2	5	6530	0.768 ± 0.053	0.008 ± 0.008
MNE	1	6	14,398	0.841 ± 0.043	0.010 ± 0.003
	2	7	19,776	0.864 ± 0.070	0.009 ± 0.003
Overall	-	43	106,987	0.831 ± 0.089	0.008 ± 0.007

**Table 2:** The effect of duration of transportation on irradiated male mosquito mortality (around 1500 sterilized males/box).

Duration of Transportation (Hours)	Destination Country	No of Shipment (Trial)	No of Males Shipped/Trial	Average No of Males (±SE)	Mortality during Transportation (%)	Average Mortality (±SE) ^a^
48 ± 2	Montenegro	1	15,000	17,400 ± 993	78.16	39.83 ± 10.01a
		2	22,000		53.31	
		3	20,000		89.38	
		4	15,000		72.65	
		5	15,000		15.83	
		6	21,000		13.72	
		7	15,000		32.92	
		8	16,000		9.41	
		9	20,000		14.55	
		10	15,000		8.39	
22 ± 2	Greece	1	15,000	13,880 ± 884	12.7	15.66 ± 2.75b
		2	18,000		16.8	
		3	15,000		15.1	
		4	19,000		24.9	
		5	12,000		5.0	
		6	15,000		10.4	
		7	17,000		24.7	

^a^ Means followed by the same letter do not differ significantly (Welch’s *t*-test, *p* = 0.05).

**Table 3:** Response (time to death) of wild and sterilized *Aedes albopictus* males under food stress conditions (only water).

Type of Male	Average Life Span (Days)	Quartiles (Days ± SE)		
		25	50	75
Wild (n = 50)	5.40 ± 0.56 a	6.00 ± 0.83	4.00 ± 0.50	3.00 ± 0.38
Sterile (n = 50)	5.98 ± 0.32 a	7.00 ± 0.35	6.00 ± 0.50	5.00 ± 0.49

Different letters indicate significant differences (pairwise comparisons, log rank test, *p* > 0.05).

**Table 4:** Response (time to death) of wild and sterilized *Aedes albopictus* males under food and water starvation.

Type of Male	Average Life Span (Hours)	Quartiles (Days ± SE)		
		25	50	75
Wild (n = 50)	36.24 ± 1.47 a	48.00 ± 1.25	36.00 ± 1.69	24.00 ± 0.00
Sterile (n = 50)	31.92 ± 1.35 b	36.00 ± 1.55	36.00 ± 1.40	24.00 ± 1.84

Different letters indicate significant differences (pairwise comparisons, log rank test, *p* < 0.05).

**Table 5:** Variables of the Cox proportional hazards model regarding effects of male type and mating on the adult lifespan.

Source of Variation	*β* ^1^	SE ^2^	Exp(β) ^3^	*p* ^4^
Male type	−3.95	0.39	0.01	<0.001
Mating status	−0.26	0.22	0.76	0.23
Mating status × Male Type	0.52	0.30	1.69	0.08

^1^*β* estimated coefficients in the Cox proportional hazards regression model, ^2^ SE standard error of the coefficients, ^3^ Exp(β) exponential of an estimated regression coefficient, ^4^ *p* value in the Cox hazard ratio analysis.

**Table 6:** Mating propensity of sterilized and wild *Aedes albopictus* males in no choice experiments involving wild virgin females.

Male Type	N	Mating (%)	Latency (min ± SE)	Duration (min ± SE)
Wild	90	55.56 a	75.18 ± 10.07 a	1.46 ± 0.20 a
Sterile	90	28.89 b	105.34 ± 15.07 a	1.64 ± 0.23 a
Test		*x*^2^ = 13.1	*t*-test = 1.704	*t*-test = 0.545
df	-	1	74	74
*p*	-	<0.001	0.093	0.587

Values in each column followed by the same letter do not different significantly *p* ≥ 0.05.

**Table 7:** Mating competitiveness of sterile and wild *Aedes albopictus*. In total 98 replicates were considered.

Male Type	N	Mating (%)	Latency (min ± SE)	Duration (min ± SE)
Wild	48	67.6 a	37.23 ± 4.38 a	0.85 ± 0.11 a
Sterile	23	32.4 b	23.22 ± 3.84 b	0.78 ± 0.07 a
Test	-	*x*^2^ = 8.8	*t*-test = 1.95	*t*-test = 0.40
df	-	1	69	69
*p*	-	<0.01	0.02	0.32

Values in each column followed by the same letter do not different significantly *p* ≥ 0.05.

## Data Availability

All data are available in the manuscript.
